# Quantitative lung tissue functional analysis for pulmonary adverse event risk assessment prior to thoracic radiotherapy

**DOI:** 10.1016/j.phro.2026.101022

**Published:** 2026-06-17

**Authors:** Sean R. Miller, Daniel Polan, Chase Hadley, Karen Vineberg, Christina Lockhart, David N. O'Dwyer, Wassim W. Labaki, Craig J. Galban, Steven G. Allen, Martha Matuszak, Shruti Jolly, Krithika Suresh, Charles K. Matrosic

**Affiliations:** a Department of Radiation Oncology, University of Michigan, Ann Arbor, MI, United States of America; b Division of Pulmonary & Critical Care Medicine, Department of Internal Medicine, University of Michigan, Ann Arbor, MI, United States of America; c Department of Radiology, University of Michigan, Ann Arbor, MI, United States of America; d Department of Biostatistics, University of Michigan, Ann Arbor, MI, United States of America

**Keywords:** Lung function imaging, Parametric response mapping, pulmonary toxicity

## Abstract

**Background and purpose:**

Pulmonary adverse events are a limiting factor in thoracic radiotherapy and more common in patients with pulmonary comorbidities. Parametric response mapping (PRM) is an effective method of providing 3-dimensional underlying lung comorbidity data. We hypothesized that a commercial PRM software could identify patients at increased risk of pulmonary adverse events during radiotherapy.

**Materials and methods:**

Paired inspiratory/expiratory computed tomography (CT) scans obtained during simulation were processed using Lung Density Analysis (LDA) to classify lung tissue as Normal, Functional Low Density (FLD), Persistent Low Density (PLD), and Inspiration High Density (IHD). Patients were classified as elevated FLD, PLD, or IHD using predetermined thresholds. Multivariable models investigated associations between grade ≥ 2 pulmonary adverse events and (i) LDA measures, and (ii) clinical lung disease history. Model discrimination was assessed using area under the receiver operating characteristic curve (AUC).

**Results:**

Among 98 patients receiving definitive-intent thoracic radiotherapy, 22 experienced grade ≥ 2 pulmonary adverse events. Elevated IHD (Odds Ratio [OR] = 2.95; 95% Confidence Interval [CI]:1.12–7.97; *p* = 0.03) and PLD (OR = 2.32; 95%CI:0.80–6.52; *p* = 0.11) were associated with increased odds of adverse events. The LDA-based multivariable model demonstrated higher, but not significantly improved, discrimination than a model with lung disease history (apparent AUC 0.768 vs. 0.735; *p* = 0.47; optimism-corrected AUC 0.705 vs. 0.686).

**Conclusion:**

Elevated IHD was a significant predictor of pulmonary adverse events, with PLD demonstrating a similar but nonsignificant trend. LDA measures demonstrated predictive performance comparable to clinical lung disease history, suggesting potential utility as a risk-screening tool pending validation in larger cohorts.

## Highlights

• Parametric Response Mapping was evaluated in 98 thoracic radiotherapy patients.
• Elevated inspiration high density increased pulmonary adverse event odds 2.95-fold.
• Clinical lung disease history achieved an area under the curve of 0.735.
• Parametric Response Mapping measures achieved an area under the curve of 0.768.
• Parametric Response Mapping may aid in pulmonary adverse event risk screening.

## Graphical abstract

**Figure:**
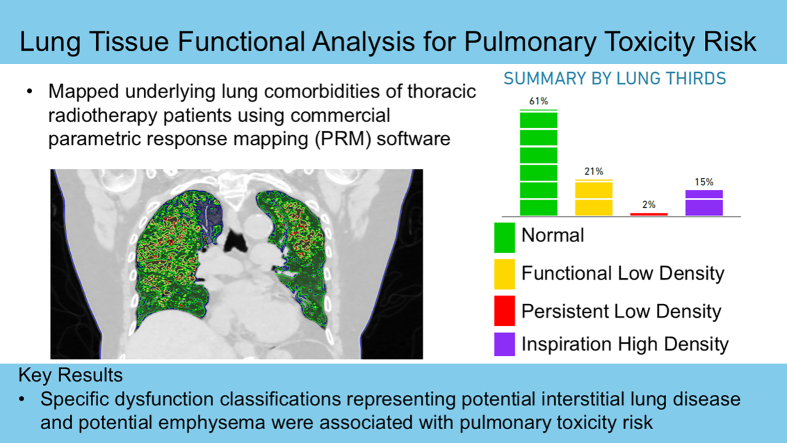

## 1 Introduction

Radiotherapy is a cornerstone of nonoperative treatment for malignancies in the chest. However, one limiting adverse event of radiation to the lung is radiation pneumonitis. Many prior attempts at predicting pulmonary adverse events relied on dose-volume histogram (DVH) data, particularly the volume of lung receiving 5 Gy or higher (V_5Gy_), 20 Gy or higher (V_20Gy_), and mean lung dose (MLD) [1], [2], [3]. However, this approach is agnostic to underlying pulmonary function.

Advances in lung function imaging in single-photon emission computed tomography (SPECT), positron emission tomography (PET), computed tomography (CT), and magnetic resonance imaging (MRI) have allowed for quantitative spatial mapping of lung tissue function and treatment planning optimization to avoid lung segments based on function [2], [4], [5], [6], [7], [8], [9], [10], [11]. CT-based lung function imaging in particular has been of interest in radiation oncology due to widespread use and availability in clinics. Retrospective studies using various approaches have shown through CT-based lung ventilation imaging of radiotherapy patients that dose to regions of functional lung is predictive of adverse event risk, which is consistent with findings using other imaging modalities [4], [8], [12], [13], [14]. Many studies suggested that reducing dose to highly ventilated regions of the lung could potentially reduce pulmonary adverse event rates beyond the simple reduction of overall lung dose metrics. Recently, published Phase II trials investigating CT-based ventilation functional lung avoidance found varied results, with some showing adverse event reductions for the entire cohort and others showing adverse event reduction only for conventionally fractionated patients and not patients receiving stereotactic body radiation therapy (SBRT) [6], [7]. Underlying pulmonary dysfunction, such as interstitial lung disease (ILD) is a well-known predictor of pulmonary adverse events [15] and additional studies have suggested that dose to dysfunctional lung may also play a role in adverse event risk [16], [17]. Preliminary evaluations of in-house developed CT-based artificial intelligence (AI) tools have proven them to be effective at identifying patients with elevated adverse event risk due to ILD, but few commercial tools exist to fulfill this role in radiation oncology clinics [18].

Parametric Response Mapping (PRM) is a lung function imaging method that has been utilized as a CT-based biomarker for underlying lung comorbidities [19], [20], [21]. PRM is performed by acquiring paired full inspiration and full expiration CT scans of the patient's lungs and deformably registering the lungs in both scans, resulting in paired Hounsfield Unit (HU) values which are mapped to the lung classifications of normal lung, small airways disease (SAD), emphysema, and parenchymal disease (PD). Unlike other CT-based approaches, such as measuring ventilation or pulmonary function, PRM identifies underlying lung comorbidities and makes distinctions between different regions of pulmonary dysfunction. PRM has been used to detect pneumonitis in bone marrow transplant patients, fibrosis in lung transplant patients, and COPD progression in smokers [19], [20], [21], [22], [23]. In this work, we investigated the utility of PRM in predicting pulmonary adverse events following thoracic radiation.

## 2 Materials and methods

### 2.1 Cohort definition and chart review

Lung Density Analysis (LDA, Imbio Inc., Minneapolis, MN), a commercial PRM software, was introduced at the University of Michigan Department of Radiation Oncology as an imaging option performed at the treating physician's discretion during treatment simulation to provide additional information about underlying lung composition. Between March 2022 and May 2023, 131 clinical treatment simulations were performed and included scans for LDA in addition to the patient's treatment planning CT. We obtained IRB approval to retrospectively identify and analyze patients treated with definitive intent, either for primary lung cancer or under an oligometastatic paradigm, for whom LDA was successfully performed, and dose data were available. Patients were excluded from analysis due to palliative intent of treatment (9 patients), poor scan quality (14 patients), poor image registration (8 patients), and/or re-simulations (8 patients). This left 98 patients for analysis. Patient data including gender, age, smoking history, diagnosis, and concurrent systemic therapy were obtained via chart review. Pulmonary adverse events were scored using the common terminology criteria for adverse events (CTCAE) v5.0 via chart review and defined as any grade 2 or higher pulmonary-related adverse event (e.g., pneumonitis, dyspnea, pleural effusion, cough, etc.). We did not restrict our review to pneumonitis and included a wider range of pulmonary toxicities as we anticipated LDA could be prognostic for a range of pulmonary toxicities that are clinically meaningful and could potentially allow for preemptive action to assist these patients, such as a timely referral to pulmonology. MLD from the delivered treatment plan was calculated for all patients in equivalent dose in 2-Gray fractions (EQD2Gy) using an α/β of 2.5 Gy.

### 2.2 LDA acquisition, registration, and analysis

**Fig. 1:**
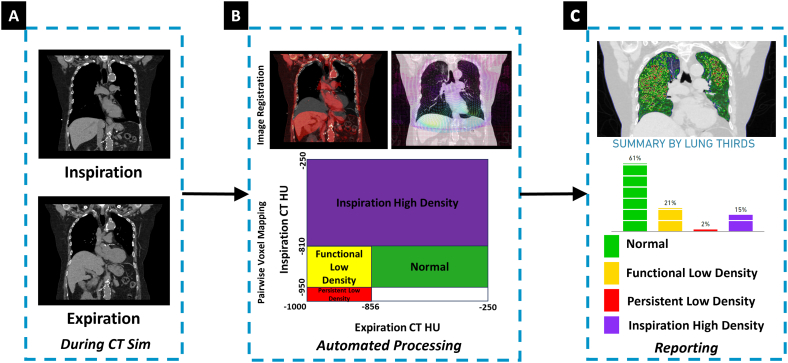
The clinical workflow implemented to acquire and process LDA scans. A) Deep inspiration and expiration computed tomography (CT) scans are acquired during CT simulation. B) CT scans are automatically processed in the LDA system. C) A report of the analysis is generated and classification maps are imported into the treatment planning system.

The clinical workflow to acquire the LDA data is shown in Fig. 1. First, paired voluntary full inspiration and full expiration (to residual volume) CT images were acquired at simulation, obtained separately from treatment planning simulation scans. Scans were acquired with a Philips Brilliance Big Bore CT or Siemens Somatom Confidence CT, depending on patient location, using 120 kVp, and a B kernel, 1-mm slice thickness reconstruction. The scans were imported into the Imbio online portal and processed through the LDA-Functional algorithm, which in an automated fashion segmented the lungs and deformably registered the scans. Inspiration scans were registered to the expiration scans, as stated by the manual (Lung Density Analysis v5.1.1 Software User Manual, Imbio Inc). The paired HUs of each voxel at inspiration and expiration were automatically recorded by the vendor algorithm to assign functional classification as previously defined by Galbán et al. [19], [21]. Voxels with ≥ − 856 HU at expiration and inspiration values between −950 HU and − 810 HU were classified as normal lung function. Functional low density (FLD) voxels were defined as expiration <−856 HU and inspiration between −950 HU and − 810 HU and was equivalent to the SAD classification presented by Galban et al. and Owen et al. [19], [24]. Persistent low density (PLD) voxels, were defined as expiration <−856 HU and inspiration <−950 HU and was equivalent to the emphysema classification presented by Galban et al. and Owen et al. [19], [24]. Inspiration high density (IHD) voxels were defined as inspiration ≥ − 810 HU and was equivalent to the PD classification presented by Belloli et al. and Owen et al. and also represent potential ILD or fibrosis [20], [24]. A report of lung classifications and a 3D visual mapping were generated for treating physicians as additional clinical information, however lung classifications were not utilized in radiation treatment planning. Physicians were notified of elevated levels of FLD, PLD, or IHD defined as ≥20%, ≥5%, ≥20% of total lung volume, respectively. These cutoff values were derived empirically from previously published values [19], [20], [21]. Note, the value provided for the IHD volume is due to CT partial volume effects at the lung edge, causing approximately 15% of the lungs to be misidentified as IHD in most patients, due to uncertainties in lung segmentation by the software, which required further visual assessment by medical physicists (Charles Matrosic and Daniel Polan) to determine if IHD levels were truly elevated [20], [21]. During this assessment, physicists were blinded to outcomes. Examples of LDA results with and without elevated IHD are presented in **Figs. S2** and **S3**.

### 2.3 Variable definition

Each patient had four LDA components (Normal, FLD, PLD, and IHD) expressed as percentages summing to 100%. Since these variables are compositional, abnormal-to-normal lung classification ratios were computed to represent relative burden of abnormal density. Binary variables were created to identify patients exceeding the pre-specified abnormality thresholds described above. History of lung disease was coded as a binary indicator (present versus absent) via chart review (i.e., COPD, ILD, asthma, or prior surgery). Positive smoking status was defined as a binary indicator of being a current smoker or ≥ 10 pack-years smoking history. Other demographic and clinical measures included age, gender, receipt of immunotherapy, receipt of chemotherapy, conventional fractionation versus SBRT, and MLD.

### 2.4 Statistical analysis

Descriptive statistics were used to summarize patient characteristics and LDA measures overall and by pulmonary adverse event status. Group comparisons were performed using Wilcoxon rank sum tests for continuous predictors and chi-squared tests for binary predictors. Univariate logistic regression models were used to assess associations between pulmonary adverse events and LDA measures, history of lung disease, and other demographic and clinical variables. Multivariable logistic regression models were fit for elevated LDA classification and history of lung disease, adjusted for pre-specified clinical variables (MLD, smoking, immunotherapy, SBRT) and factors associated with pulmonary adverse events in univariate analysis (*p* < 0.1). Model discrimination was assessed using area under the receiver operating characteristic curve (AUC) with corresponding 95% confidence intervals (CIs) estimated via bootstrapping. AUCs were compared using the DeLong test. Internal validation was performed using bootstrap-derived optimism-corrected AUCs. Sensitivity analyses used Cox proportional hazards models and Harrell's concordance index (C-index) to account for variable follow-up time and censoring. Associations between elevated LDA classifications and pulmonary adverse event type (pneumonitis, non-pneumonitis, no pulmonary adverse event) were explored using Fisher's exact tests. Analyses were performed using R 4.5.2, and statistical significance was assessed at a significance level of 0.05.

## 3 Results

From March 2022 to May 2023, 98 patients underwent definitive thoracic radiation with LDA performed during simulation. Median age was 71 years, 49% of patients were women, and nearly all patients had Karnofsky Performance Status ≥70 (Table 1). At baseline, 46% of patients had past history of lung disease (82% COPD, 9% asthma, 9% ILD, and 4% prior lung surgery). With a median follow-up time of 8.9 months, 22 patients experienced grade ≥ 2 pulmonary adverse events.

**Table 1:** Patient characteristics.

Characteristic	Overall, *N* = 98^*1*^	No RILT, *N* = 76^*1*^	RILT, *N* = 22^*1*^	p-value^*2*^
Age	71 (65,77)	72 (66,77)	69 (64,75)	0.49
Gender				0.005
Female	48 (49%)	43 (57%)	5 (23%)	
Male	50 (51%)	33 (43%)	17 (77%)	
KPS				0.19
60	2 (2.1%)	2 (2.7%)	0 (0%)	
70	23 (24%)	16 (22%)	7 (33%)	
80	25 (26%)	17 (23%)	8 (38%)	
90	32 (34%)	29 (39%)	3 (14%)	
100	13 (14%)	10 (14%)	3 (14%)	
Unknown	3	2	1	
Baseline Lung Disease	45 (46%)	32 (42%)	13 (59%)	0.16
Smoker				0.34
Never	13 (13%)	10 (13%)	3 (14%)	
Former	65 (66%)	48 (63%)	17 (77%)	
Current	20 (20%)	18 (24%)	2 (9.1%)	
Pack Years	30 (10,46)	28 (10,46)	30 (18,60)	0.41
Number of Prior Courses of Radiation				0.36
0	72 (73%)	58 (76%)	14 (64%)	
1	23 (23%)	16 (21%)	7 (32%)	
2	3 (3.1%)	2 (2.6%)	1 (4.5%)	
Lung Primary				0.99
No	8 (8.2%)	6 (7.9%)	2 (9.1%)	
Yes	67 (68%)	52 (68%)	15 (68%)	
Empiric Radiation	23 (23%)	18 (24%)	5 (23%)	
Stage				0.74
Early stage NSCLC	53 (55%)	43 (57%)	10 (48%)	
Limited-stage SCLC	6 (6.2%)	5 (6.6%)	1 (4.8%)	
Locally advanced NSCLC	18 (19%)	12 (16%)	6 (29%)	
Metastatic	2 (2.1%)	2 (2.6%)	0 (0%)	
Oligometastatic	18 (19%)	14 (18%)	4 (19%)	
Unknown	1	0	1	
Chemotherapy	24 (24%)	17 (22%)	7 (32%)	0.36
Immunotherapy	15 (15%)	11 (14%)	4 (18%)	0.74
Definitive Treatment				0.97
Conventional Radiation	36 (37%)	28 (37%)	8 (36%)	
SBRT	62 (63%)	48 (63%)	14 (64%)	

^*1*^ Median (IQR); n (%).

^*2*^ Wilcoxon rank sum test; Pearson's Chi-squared test; Fisher's exact test.

The median percentages of normal lung, FLD, PLD, and IHD were 58% (interquartile range [IQR] 45–67%), 18% (IQR 6–30%), 1% (IQR 0–4%), and 17% (IQR 12–22%), respectively. A histogram of the distribution of the percentage of lung LDA categorization is shown in **Fig. S1**. All patients had trace amounts of IHD upwards of approximately 15% of the lung volume due to partial volume artifacts at the edge of the lung and lung vessels being misidentified as IHD.

In univariate logistic regression analyses (Table 2), continuous ratios of abnormal-to-normal classification were not significantly associated with pulmonary adverse events. Exploratory analyses suggested non-linear relationships, which likely reflected limited sample sizes at the ratio distribution extremes as well as physiological limits to certain lung classifications. In binary comparisons of elevated versus non-elevated abnormal classifications, elevated IHD (Odds Ratio [OR] = 2.95; 95%CI:1.12–7.97; *p* = 0.030), defined as exceeding 20% of the lung volume, was significantly associated with increased odds of pulmonary adverse events, elevated PLD (OR = 2.32; 95%CI:0.80–6.52; *p* = 0.11), defined as exceeding 5% of the lung volume, exhibited a similar but nonsignificant trend, and elevated FLD (OR = 0.71; 95%CI:0.26–1.85; *p* = 0.49) was not significantly associated with pulmonary adverse events. Among clinical variables, gender was the only factor associated with pulmonary adverse events at a pre-specified threshold (*p* < 0.1). Of note, males were more likely to have a history of lung disease (58% vs. 33%; *p* = 0.014) and to receive immunotherapy (22% vs. 8%; *p* = 0.06) (**Table S1**). Other pre-specified covariates considered for multivariable adjustment, including MLD, smoking status, SBRT, and immunotherapy, were not significantly associated with pulmonary adverse events in univariate analyses. To limit model complexity given the limited number of events, subsequent multivariable models were adjusted for gender, MLD, and receipt of SBRT. A post-hoc sensitivity analysis was performed to fit a more parsimonious multivariable model that adjusted only for gender.

**Table 2:** Univariate logistic regression model results investigating the association of each variable with grade 2+ lung adverse events.

Variable	Odds Ratio (95% CI)	p-value
FLD %/Normal %	0.86 (0.33–1.75)	0.71
PLD %/Normal %	0.83 (0.17–1.85)	0.73
IHD %/Normal %	1.59 (0.22–9.02)	0.60
FLD Elevated	0.71 (0.26–1.85)	0.49
PLD Elevated	2.32 (0.80–6.52)	0.11
IHD Elevated	2.95 (1.12–7.97)	0.030
Male (vs Female)	4.43 (1.57–14.61)	0.008
Age (years)	0.98 (0.93–1.03)	0.49
Mean Lung Dose (EQD2Gy)	1.05 (0.94–1.18)	0.35
SBRT	1.02 (0.39–2.84)	0.97
Recent Smoker	1.11 (0.35–4.25)	0.87
Diagnosed Lung Disease	1.99 (0.76–5.36)	0.16
Chemotherapy	1.62 (0.54–4.53)	0.37
Immunotherapy	1.31 (0.33–4.37)	0.67

Multivariable logistic regression analyses are summarized in Table 3. Based on univariate findings, primary multivariable models focused on IHD and PLD components. Model 1, which included a priori specified clinical covariates and significant LDA variables from univariate analysis, demonstrated elevated IHD (OR = 3.58, 95%CI:1.17–11.9; *p* = 0.029) and PLD (OR = 3.45, 95%CI:0.99–13.0; *p* = 0.06) were independently associated with increased odds of pulmonary adverse events after adjustment for gender, MLD, and SBRT. Model 2, which included only clinical covariates, identified a positive, but not statistically significant, association between history of lung disease and pulmonary adverse events (OR = 1.55, 95%CI:0.56–4.43; *p* = 0.40). In Model 3 (combined LDA and lung disease history), elevated IHD remained significantly associated with pulmonary adverse events, while history of lung disease did not meaningfully alter observed associations. Across models, discrimination (Table 4) was highest for models incorporating LDA components. Model 1 demonstrated higher apparent performance, although the difference was not statistically significant compared with Model 2 (AUC = 0.768 vs. 0.735; *p* = 0.47). Optimism-corrected (OC) AUCs were attenuated but remained highest for Model 1 (OCAUC = 0.705). This model had improved performance compared with models that included each component alone (OCAUC: IHD = 0.697; PLD = 0.692, not shown) and similar performance to Model 3 that combined LDA components with history of lung disease (OCAUC = 0.702). Results were consistent in the sensitivity analyses using a parsimonious model adjusting for gender only (**Table S2**) and in a time-to-event framework (**Table S3**). In multivariable models for LDA measures, elevated IHD remained significantly associated with adverse events. In Cox proportional hazards models, the association between elevated PLD and adverse events reached statistical significance. Models with LDA measures demonstrated improved discrimination compared to models including lung disease history.

**Table 3:** Multivariable logistic regression models assessing the association of clinical and LDA variables with grade 2+ lung adverse events adjusted for gender, mean lung dose, and receipt of SBRT. Model 1 includes binary classification of LDA components indicating patients having elevated levels of abnormal classification, Model 2 includes a binary indicator of the impact of a prior clinical diagnosis of lung disease, and Model 3 includes both the indicator of elevated levels of abnormal classifications and a clinical diagnosis of lung disease.

Variable	Model 1 Odds Ratio (95% CI, p-value)	Model 2 Odds Ratio (95% CI, *p*-value)	Model 3 Odds Ratio (95% CI, p-value)
PLD Elevated	3.45 (0.99–12.95, p = 0.06)		3.26 (0.89–12.66, *p* = 0.087)
IHD Elevated	3.58 (1.17–11.93, p = 0.029)		3.55 (1.16–11.84, p = 0.030)
Male (vs Female)	4.06 (1.36–14.08, *p* = 0.017)	4.39 (1.49–15.12, *p* = 0.011)	3.87 (1.25–13.75, *p* = 0.025)
Mean Lung Dose (EQD2Gy)	1.15 (0.95–1.41, *p* = 0.17)	1.14 (0.96–1.37, *p* = 0.15)	1.15 (0.95–1.41, *p* = 0.16)
SBRT	2.62 (0.51–17.64, *p* = 0.28)	2.28 (0.51–12.67, *p* = 0.31)	2.64 (0.51–17.55, p = 0.28)
Diagnosed Lung Disease		1.55 (0.56–4.43, p = 0.40)	1.23 (0.39–3.85, *p* = 0.72)

**Table 4:** Apparent and optimism-corrected area under the receiver operating characteristic curve (AUC) for adverse event prediction models.

Model predictors 1, 2	AUC (95% CI)	Optimism-corrected AUC
Model 1 (LDA components)	0.768 (0.658–0.878)	0.705
Model 2 (lung disease history)	0.735 (0.602–0.868)	0.686
Model 3 (combined LDA and physician-graded)	0.776 (0.669–0.883)	0.702

1 Models are adjusted for Gender, Mean Lung Dose (EQD2Gy), SBRT;

2 All LDA models include the components as binary indicators of elevated levels of the abnormal classification.

In the exploratory analysis shown in Table 5, we examined the relationship between elevated LDA classifications and type of pulmonary adverse events, 80% of patients that developed pneumonitis had elevated levels of IHD compared to 44% of those with non-pneumonitis pulmonary adverse events and 30% with no pulmonary adverse events (*p* = 0.051). Among patients that developed non-pneumonitis pulmonary adverse events (dyspnea, hypoxia, lung infection, etc.), 50% had elevated levels of PLD compared to 0% of those with pneumonitis and 19% of those with no pulmonary adverse events (*p* = 0.024).

**Table 5:** Association between elevated levels of LDA classifications and type of pulmonary adverse event, categorized as pneumonitis, non-pneumonitis, or no pulmonary adverse event.

Variable	Non-pneumonitis Pulmonary Adverse Event *N* = 16	Pneumonitis *N* = 5	No Pulmonary Adverse Event *N* = 77	p-value
FLD Elevated	8 (50%)	0 (0%)	34 (44%)	0.12
PLD Elevated	8 (50%)	0 (0%)	15 (19%)	0.024
IHD Elevated	7 (44%)	4 (80%)	23 (30%)	0.051

## 4 Discussion

We performed a retrospective analysis of a quantitative lung comorbidity mapping method prior to thoracic radiation. Elevated baseline IHD was significantly associated with pulmonary adverse events. Elevated PLD demonstrated a similar but not statistically significant association in the primary analysis but reached statistical significance in a sensitivity time-to-event analysis. These findings suggested that the commercial PRM software could be used as a quantitative method, easily deployed at time of simulation, to identify patients at elevated risk of pulmonary adverse events following thoracic radiation.

Continuous changes of baseline abnormal-to-normal classification ratios were not significantly associated with adverse events. This suggested a threshold may exist at which pulmonary burden of these classifications significantly relates to pulmonary adverse event risk. As expected in a population with significant smoking history and other comorbid lung conditions, our cohort showed higher IHD and FLD percentages than reported for healthy control subjects by Galban et al., which were 11% and 8.4%, respectively [21], [25], [26], [27].

There is a well-known relationship between ILD and propensity for adverse events [15], [28], [29], [30], [31], but specific quantifications and thresholds of the lung volume impacted by disease have not been reported previously, to the authors' knowledge. These findings and potential clinical use case are similar to those of Bacon et al., who screened for potential ILD in a non-small cell lung cancer cohort by evaluating patient treatment planning CTs with an AI-based model [18] They found a specific screening-score threshold was significantly associated with an elevated lung adverse event risk, informing physicians of which patients need closer observation and could benefit from further ILD evaluation. PRM may serve a similar function by using an available commercial tool to evaluate patients for potential undiagnosed or subclinical disease at time of simulation and predict adverse events.

Additionally, we utilized a broader pulmonary adverse event definition, capturing significant pulmonary events potentially unrelated to radiation, like dyspnea, hypoxia, and lung infection, that could have been due to underlying comorbid conditions such as COPD. PRM has previously been shown to effectively classify patients' COPD severity [19]. Other CT-based methods have also been shown to have high discriminatory ability to identify patients at high risk of COPD-exacerbations [32]. The PLD classification is associated with COPD, and in exploratory analysis, was associated with non-pneumonitis adverse events, some of which may have been related to underlying COPD. Furthermore, IHD is associated with ILD and nearly all patients that developed pneumonitis had elevated IHD. As both types of adverse events are clinically meaningful, PRM may have the added utility of predicting the type of adverse event a patient may develop, which have different clinical courses and treatments. If patients have elevated PLD and are not already evaluated for COPD or under the care of a pulmonologist, then the treating radiation oncologist can be alerted to refer based on the PRM results. Likewise, elevated IHD should lower the threshold to consider workup and treatment of pneumonitis if symptoms develop after radiation treatment.

Our study has several limitations. First, this analysis was retrospective and included patients treated with different indications, doses, and fractionations. While this heterogeneity may introduce confounding, it allowed for a broader baseline lung function range, particularly as some SBRT patients had metastatic disease and therefore were less likely to have baseline lung dysfunction. Second, modest sample size limited statistical power for detecting small effect sizes, formal interaction tests, and non-linear relationships. The number of events relative to the number of predictors was limited, which increases the risk of overfitting and reduces model stability. Accordingly, model complexity was constrained to mitigate overfitting, and internal validation was performed using bootstrap-based optimism correction. While results were consistent across sensitivity analyses, validation in larger, independent cohorts is required to assess generalizability and utility of these findings. Third, automated identification of IHD may be susceptible to misclassification due to CT partial volume effect artifacts at lung boundaries. This uncertainty was caused by the lung segmentation algorithm in the PRM software, causing some edge voxels to include non-lung parenchyma and be identified as high density, increasing the HU. To mitigate this, cases classified as elevated IHD underwent qualitative review to confirm the true presence of IHD. However, some degree of measurement uncertainty remained and should be considered when interpreting results.

In conclusion, the commercial PRM software is a promising quantitative method of classifying underlying lung comorbidities. It potentially has value in identifying patients with subclinical ILD or undiagnosed COPD who may be at higher risk for pulmonary adverse events. Further work is necessary to better understand the relationship between dose and lung classifications on adverse event risk to help guide improved treatment planning techniques.

## CRediT authorship contribution statement

**Sean R. Miller:** Writing – review & editing, Writing – original draft, Methodology, Investigation, Formal analysis, Data curation, Conceptualization. **Daniel Polan:** Writing – review & editing, Methodology, Investigation, Formal analysis, Conceptualization. **Chase Hadley:** Methodology, Investigation, Formal analysis. **Karen Vineberg:** Methodology, Investigation. **Christina Lockhart:** Methodology, Investigation. **David N. O'Dwyer:** Writing – review & editing, Conceptualization. **Wassim W. Labaki:** Writing – review & editing, Conceptualization. **Craig J. Galban:** Writing – review & editing, Methodology, Conceptualization. **Steven G. Allen:** Writing – review & editing, Conceptualization. **Martha Matuszak:** Writing – review & editing, Methodology, Conceptualization. **Shruti Jolly:** Writing – review & editing, Conceptualization. **Krithika Suresh:** Writing – review & editing, Writing – original draft, Methodology, Investigation, Formal analysis, Data curation, Conceptualization. **Charles K. Matrosic:** Writing – review & editing, Writing – original draft, Project administration, Methodology, Investigation, Formal analysis, Data curation, Conceptualization.

## Declaration of competing interest

The authors declare the following financial interests/personal relationships which may be considered as potential competing interests: (This research received no specific grant funding from any funding agency in the public, commercial, or not-for-profit sectors. Daniel Polan receives research funding from Varian Medical Systems. Wassim Labaki, has received grants from the National Institutes of Health, has been a paid consultant for Inogen and Verona, received travel funding and an honorarium from Inogen, and is the DSMB Executive Secretary for a pilot trial on aspirin in COPD conducted at Johns Hopkins University. Craig Galban is a co-inventor of Parametric Response Mapping, which the University of Michigan licensed to 4D Medical. Martha Matuszak received grant funding and meeting travel support from 10.13039/100007210Varian Medical Systems and has a licensing agreement with Fuse Oncology. Shruti Jolly has received honoraria from Varian Medical Systems and AstraZeneca and is a board member of the Michigan Society of Heme Onc. Charles Matrosic has received an honorarium from Varian Medical Systems and travel funding from ASTRO. All other authors have no relevant conflicts of interest to disclose.)
