# Supplementary material for: Quantitative lung tissue functional analysis for pulmonary adverse event risk assessment prior to thoracic radiotherapy
Source: Phys Imaging Radiat Oncol. 2026 Jun 17;40:101022. doi: 10.1016/j.phro.2026.101022 (PMC13324496; doi:10.1016/j.phro.2026.101022)
Supplement: Supplementary file 1 — Supplementary material [file mmc1.pdf]

Supplementary Materials for Quantitative Lung Tissue Functional Analysis for Radiotherapy Adverse Event Risk Assessment

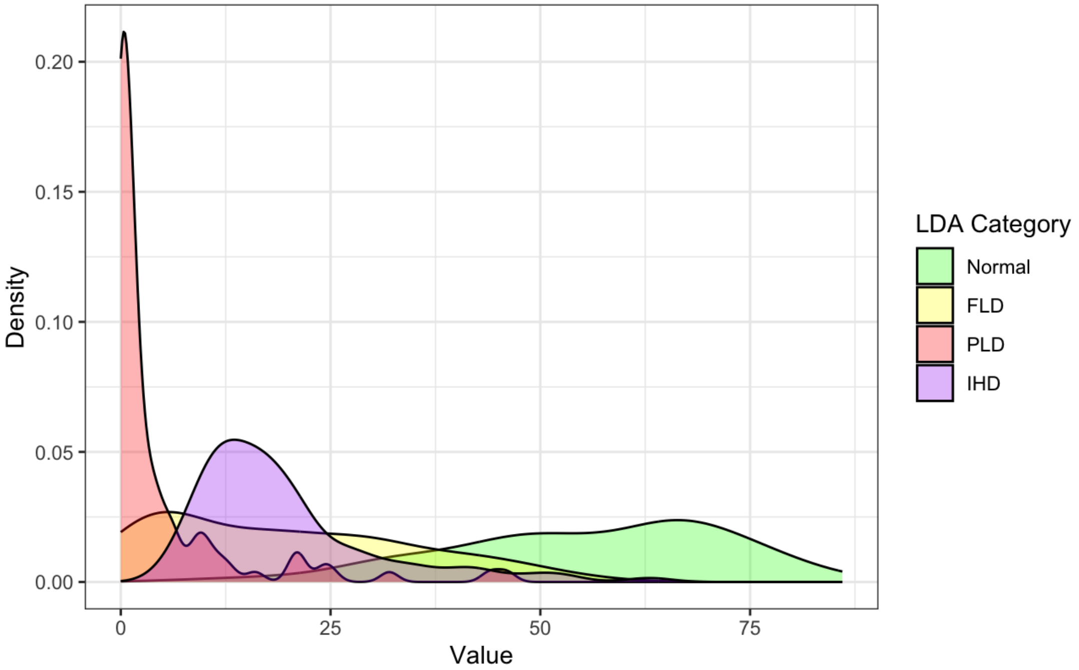

Figure S1. A histogram showing the distribution of the percentage of total lung volume of each lung voxel classification.

Table S1: Patient characteristics by gender

**Characteristic**	**Female**<br>**N = 48**[1]	**Male**<br>**N = 50**[1]	**p-value**[2]
Age	72 (68,77)	70 (64,77)	0.81
KPS			0.73
60	2 (4.4%)	0 (0%)	
70	10 (22%)	13 (26%)	
80	11 (24%)	14 (28%)	
90	15 (33%)	17 (34%)	
100	7 (16%)	6 (12%)	
Unknown	3	0	
Lung Disease	16 (33%)	29 (58%)	0.014
Smoker			0.26
Never	8 (17%)	5 (10%)	
Former	28 (58%)	37 (74%)	
Current	12 (25%)	8 (16%)	
Smoker Status			0.39
Negative	11 (23%)	8 (16%)	
Positive	37 (77%)	42 (84%)	
Pack Years	27 (9,45)	30 (15,51)	0.28
Number of Prior Courses of Radiation			0.55
0	33 (69%)	39 (78%)	
1	13 (27%)	10 (20%)	
2	2 (4.2%)	1 (2.0%)	
Lung Primary			0.30
No	4 (8.3%)	4 (8.0%)	
Yes	36 (75%)	31 (62%)	
Empiric Radiation	8 (17%)	15 (30%)	
Stage			0.88
Early stage NSCLC	25 (52%)	28 (57%)	
Limited-stage SCLC	4 (8.3%)	2 (4.1%)	
Locally advanced NSCLC	8 (17%)	10 (20%)	
Metastatic	1 (2.1%)	1 (2.0%)	
Oligometastatic	10 (21%)	8 (16%)	
Unknown	0	1	
Chemotherapy	9 (19%)	15 (30%)	0.20
Immunotherapy	4 (8.3%)	11 (22%)	0.06
Definitive Treatment			0.88
Conventional Radiation	18 (38%)	18 (36%)	
SBRT	30 (63%)	32 (64%)	
FLD Elevated	17 (35%)	25 (50%)	0.14
PLD Elevated	9 (19%)	14 (28%)	0.28
IHD Elevated	15 (31%)	19 (38%)	0.48
Adverse Event	5 (10%)	17 (34%)	0.005

[1] Median (Minimum,Maximum); n (%)

[2] Wilcoxon rank sum test; Fisher's exact test; Pearson's Chi-squared test

Table S2: Multivariable logistic regression models assessing the association of clinical and LDA variables with grade 2+ lung adverse event adjusted for gender. Model 4 includes binary classification of LDA components indicating patients having elevated levels of abnormal classification, Model 5 includes a binary indicator of the impact of a prior clinical diagnosis of lung disease, and Model 6 includes both the indicator of elevated levels of abnormal classifications and a clinical diagnosis of lung disease. Discrimination results are presented for apparent and optimism-corrected area under the receiver operating characteristic curve (AUC).

**Variable**	**Model 4 Odds Ratio (95% CI, p-value)**	**Model 5 Odds Ratio (95% CI, p-value)**	**Model 6 Odds Ratio (95% CI, p-value)**
PLD Elevated	3.07 (0.92–10.82, p=0.07)		2.92 (0.82–10.90, p=0.10)
IHD Elevated	3.81 (1.28–12.35, p=0.019)		3.78 (1.27–12.27, p=0.020)
Male (vs Female)	3.92 (1.33–13.33, p=0.018)	4.05 (1.40–13.61, p=0.014)	3.77 (1.23–13.30, p=0.026)
Diagnosed Lung Disease		1.48 (0.54–4.18, p=0.45)	1.15 (0.36–3.59, p=0.80)
AUC (95% CI)	*0.753 (0.643–0.862)*	*0.691 (0.573–0.810)*	*0.756 (0.645–0.867)*
Optimism-corrected AUC	*0.712*	*0.666*	*0.695*

Table S3: Cox proportional hazards models assessing the association of clinical and LDA variables with time to grade 2+ lung adverse event adjusted for gender, mean lung dose, and receipt of SBRT (Models 1-3) and gender only (Models 4-6). Models 1 and 4 include binary classification of LDA components indicating patients having elevated levels of abnormal classification, Models 2 and 5 include a binary indicator of the impact of a prior clinical diagnosis of lung disease, and Models 3 and 6 include both the indicator of elevated levels of abnormal classifications and a clinical diagnosis of lung disease. Discrimination results are presented for apparent and optimism-corrected area under the receiver operating characteristic curve (AUC).

**Variable**	**Model 1 Hazard Ratio (95% CI, p-value)**	**Model 2 Hazard Ratio (95% CI, p-value)**	**Model 3 Hazard Ratio (95% CI, p-value)**	**Model 4 Hazard Ratio (95% CI, p-value)**	**Model 5 Hazard Ratio (95% CI, p-value)**	**Model 6 Hazard Ratio (95% CI, p-value)**
PLD Elevated	2.90 (1.08–7.78, p=0.035)		2.96 (1.04–8.43, p=0.042)	2.72 (1.05–7.07, p=0.040)		2.89 (1.02–8.15, p=0.045)
IHD Elevated	2.79 (1.12–6.96, p=0.028)		2.82 (1.12–7.13, p=0.028)	3.03 (1.23–7.46, p=0.016)		3.10 (1.24–7.77, p=0.016)
Male (vs Female)	2.56 (0.91–7.19, p=0.07)	3.11 (1.10–8.81, p=0.032)	2.61 (0.89–7.66, p=0.08)	2.51 (0.90–7.01, p=0.08)	2.96 (1.04–8.38, p=0.041)	2.64 (0.90–7.77, p=0.08)
Mean Lung Dose (GyEQD2)	1.08 (0.92–1.26, p=0.36)	1.08 (0.94–1.25, p=0.26)	1.08 (0.92–1.26, p=0.37)			
SBRT	1.35 (0.35–5.19, p=0.67)	1.50 (0.42–5.33, p=0.54)	1.35 (0.35–5.23, p=0.67)			
Diagnosed Lung Disease		1.31 (0.54–3.20, p=0.56)	0.93 (0.34–2.57, p=0.90)		1.28 (0.52–3.14, p=0.59)	0.86 (0.31–2.36, p=0.77)
Harrell's C-index (95% CI)	*0.734 (0.630–0.837)*	*0.684 (0.565–0.804)*	*0.734 (0.629–0.838)*	*0.717 (0.609–0.825)*	*0.646 (0.537–0.755)*	*0.708 (0.590–0.826)*
Optimism-corrected Harrell's C-index	*0.669*	*0.626*	*0.654*	*0.673*	*0.615*	*0.645*

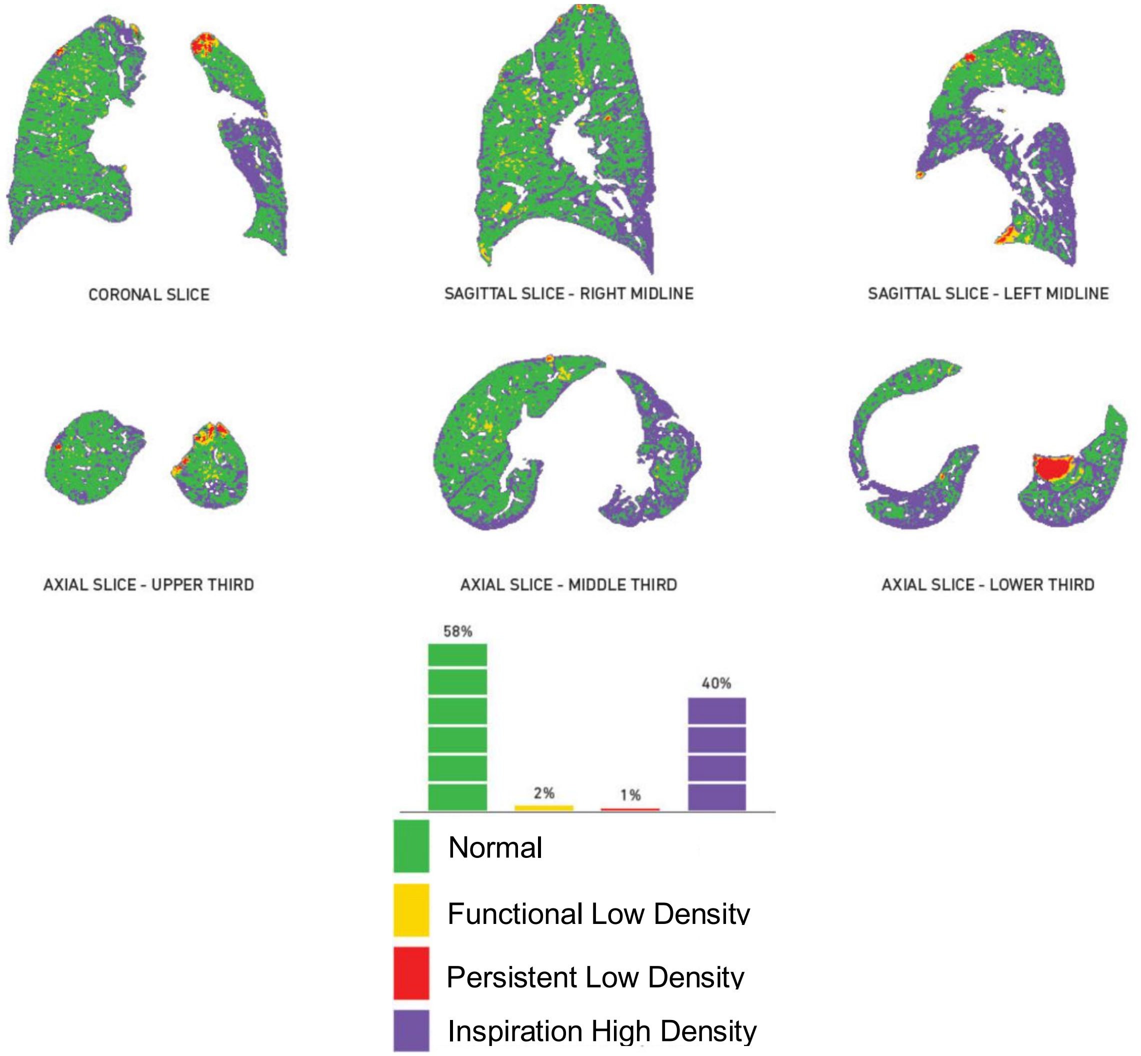

Figure S2: Example of the results of LDA for a patient with elevated Inspiration High Density.

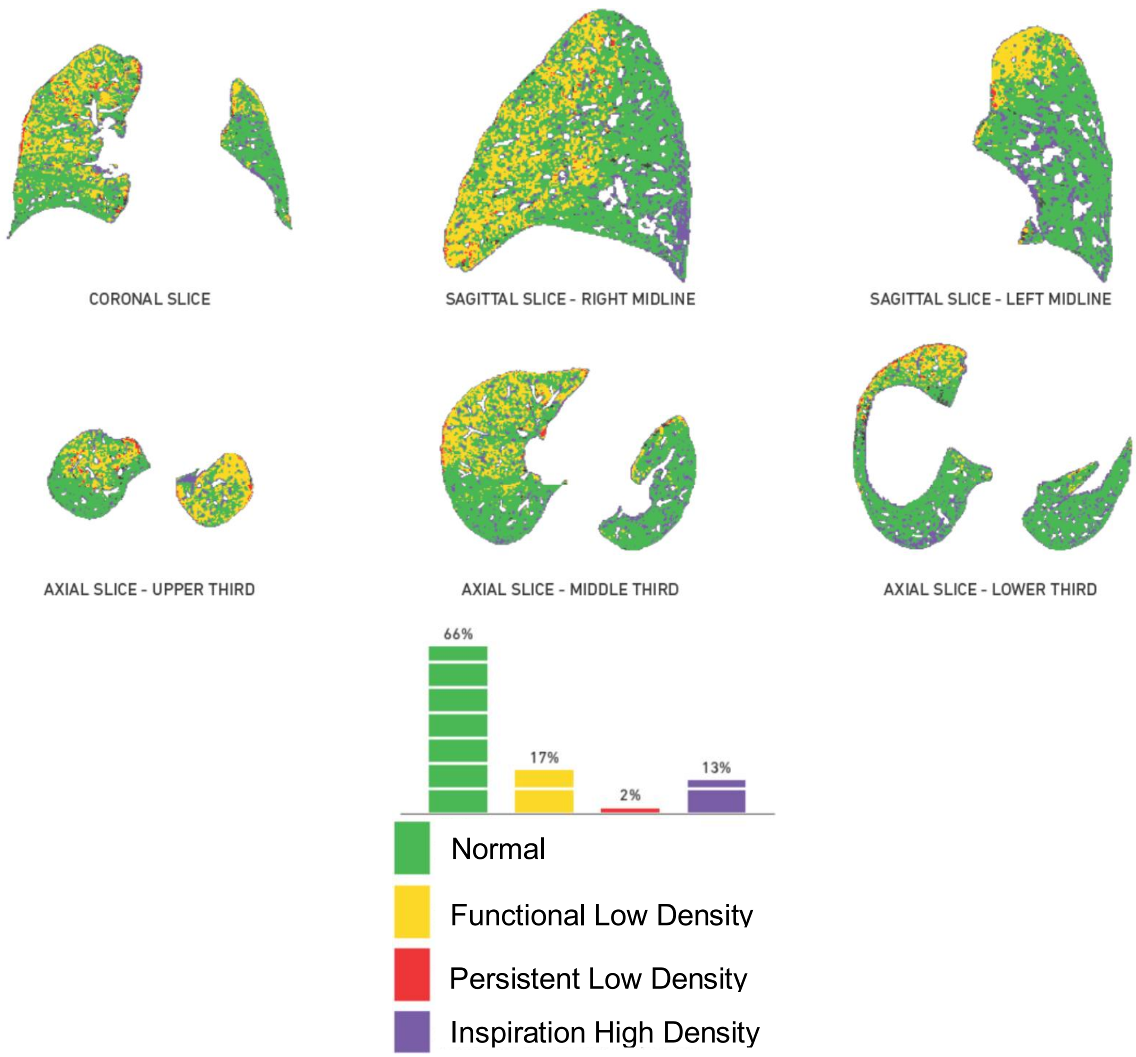

Figure S3: Example of the results of LDA for a patient with typical levels Inspiration High Density due to partial volume effects near lung borders.
